# First-time traumatic anterior shoulder dislocation: Approach for the primary health care physician

**DOI:** 10.4102/safp.v65i1.5744

**Published:** 2023-06-26

**Authors:** Ntambue Kauta, James Porter, Mubashir A. Jusabani, Stefan Swanepoel

**Affiliations:** 1 Division of Orthopaedic Surgery, Department of Surgery, Faculty of Health Sciences, University of Cape Town, Cape Town, South Africa; 2 Department of Orthopaedic Surgery, Mitchels Plain Hospital, Metro Health Services, Western Cape Government: Health and Wellness, Cape Town, South Africa; 3 Division of Family Medicine, Department of Family, Community and Emergency Care, Faculty of Health Sciences, University of Cape Town, Cape Town, South Africa; 4 False Bay Hospital, Metro Health Services, Western Cape Government: Health and Wellness, Cape Town, South Africa

**Keywords:** primary health care, shoulder, anterior dislocation, traumatic, treatment, immobilisation

## Abstract

Traumatic anterior shoulder dislocation is a very common injury encountered in emergency rooms as well as in the primary health care physician’s office. This injury occurs either in the setting of competitive or recreational sports injuries or as a high-energy injury during a fall or a road traffic accident. Common complications such as a recurrent dislocation can be predicted, monitored and prevented. Early appropriate treatment of associated cuff tears or fractures is associated with improved outcomes. There is a plethora of literature on the assessment and management of the primary anterior shoulder dislocation in specialised fields such as sports medicine, orthopaedic surgery and shoulder surgery. These studies are often highly technical, addressed to a particular subset of readers and often deal with one aspect of the management of the injury. This narrative aims to provide the reader with a simplified, evidence-based assessment and management approach for the first-time acute anterior shoulder dislocation. Emphasis is on closed reduction techniques, position and duration of immobilisation, and return to activities of life or sports. Risk factors for recurrence and other indications for primary referral to the orthopaedic surgeon are discussed. Other forms of shoulder instability such as posterior shoulder dislocation, inferior dislocation and multidirectional instability will not be the focus of this narrative.

## Introduction

Anterior shoulder dislocation is the most common joint dislocation in the human body.^1^ The shoulder’s anatomy allows for greater flexibility with inherent instability as the trade-off. The recurrence rate after an adequate initial conservative management varies from 72% to 100% in the adolescent population group.^2^ Delayed surgical management of a recurrent shoulder dislocation will lead to the development of secondary osteoarthritis of the shoulder in a very young patient. Surgical options for such a complication are limited and results often shortlived.^3^ Rotator cuff tears are another common injury associated with anterior shoulder dislocation, with the rate varying between 7% and 32% commonly affecting patients older than 40 years and rising with advanced age.^4^

During the index assessment, attention is paid to determining the mechanism of injury (traumatic vs spontaneous or recurrent), excluding associated fractures (greater tuberosity, coracoid process and glenoid fractures) and frank shoulder weakness which could be the result of a rotator cuff tear or a neurological injury. Risk factors for a recurrent dislocation must also be assessed at the initial presentation. Patients with associated fractures, shoulder weakness or risk factors for recurrent dislocation are best referred for follow-up with an orthopaedic surgeon.

## Assessment and management on the day of injury

Patients who have sustained an anterior shoulder dislocation as a result of a high-energy trauma must be initially assessed according to the advanced trauma life support principles. The purpose of this detailed examination is to exclude life-threatening injuries (cervical spine and thoracoabdominal injuries) which may be associated with the dislocation. Examination of the dislocated shoulder begins after excluding all potentially life-threatening injuries.

Consistent clinical signs of the anteriorly dislocated shoulder include:

- Patients walk with their injured shoulder dropped, slightly abducted, externally rotated and being supported by the contralateral limb.
- Loss of deltoid contour (square shoulder).
- Loss of deltopectoral groove definition (or fullness of the deltopectoral groove).
- The humeral head is palpated under the deltopectoral groove.
- Emptiness of the glenoid fossa on palpation.

The neurovascular status of the injured upper limb is assessed before and after the reduction manoeuvre. Axillary nerve neuropraxia is the most common neurological deficit found in anterior shoulder dislocation.^5^ Sensory alteration over the affected deltoid area indicates an axillary nerve injury (commonly neuropraxia). Axillary nerve motor function in the acutely injured limb is assessed by palpating for posterior deltoid contraction while asking the patient to gently extend the shoulder against resistance.

At this initial assessment, before making any attempt to relocate the shoulder, a brachial plexus assessment is conducted, assessing sensory changes and motor function by asking the patient to flex their elbow (C5 and C6 roots), extend their elbow (controlled elbow extension C7 as opposed to gravity extension), extend or dorsiflex their wrist (C6 root), and abduct as well as adduct their fingers (C8 and T1 roots).

It is crucial to perform the neurovascular assessment before and after joint relocation to be able to distinguish neurological fallout caused by the injury from manipulation manoeuvres.

Plain radiographs of the injured shoulder will complete the assessment. It is imperative to obtain the full trauma X-ray series for an injured shoulder. These include a true anteroposterior view (Grashey view), a scapula Y view (lateral or outlet view) and a modified axillary view (a true axillary view requires shoulder abduction which is not possible in an injured shoulder).

### Anteroposterior view

The humeral head is seen medial and slightly inferior to the glenoid. Greater tuberosity fractures are easily detectable and glenoid fractures may be detected. If the humeral head appears as a light bulb (light bulb sign) because of its fixed internal rotation, one should suspect a posterior dislocation that can be confirmed based on other views.

### Lateral view (scapula Y view)

The humeral head is noted anterior to the glenoid, coracoid fractures may be detected on this view.

### Axillary view

This view confirms the direction of the dislocation and coracoid process fractures are best detected on this view.

Point-of-care ultrasound imaging is becoming increasingly available in certain emergency centres and may be used by skilled physicians for the diagnosis of shoulder dislocation and assessment of the reduction. Gottlieb et al. reported a high sensitivity and specificity of point-of-care ultrasound for the assessment of shoulder dislocations.^6^

## Closed reduction techniques for anterior shoulder dislocation

The literature lists up to 23 different techniques and 17 modifications of these techniques for a closed reduction of an anterior shoulder dislocation.^7,8,9^ The most commonly used techniques are summarised in Table 1.^7,10^ Most of these techniques require some form of a pre-reduction pain control strategy varying from a parenteral analgesia or procedural sedation to regional anaesthetic blocks and intraarticular blocks. An ideal method of reduction should be simple, easily reproducible, relatively painless and can be implemented unassisted without sedation or anaesthesia with minimal or no further complications.^9^

**TABLE 1:** Summary of the different techniques for reduction of anterior shoulder dislocation.

Technique		Sedation
**Supine position, traction-based techniques**		
Traction or countertraction	Axial traction is applied to the affected extremity Counter traction is applied by an assistant using a sheet around the patient’s upper torso	Yes
Milch technique	Patient supine, upper extremity abducted and externally rotated, traction and thumb pressure is applied to push the humeral head into place	Yes
Hippocratic technique	One foot is placed in the axilla and gentle internal and external rotation with axial traction on the affected extremity	Yes
FARES (fast, reliable and safe)	Arm adducted, employ gentle vertical oscillatory movement, gradual abduction with traction is applied	Yes
Spaso technique	Scapula stabilised against stretcher, upward traction as well as external rotation applied	Yes
**Supine position, no traction technique**		
Kocher technique	Patient adducts the affected arm and flexes the elbow, the examiner grasps the wrist and gently externally rotates the shoulder until resistance is met, then forward elevates as far as possible and internal rotation of the shoulder completes the manoeuvre	Yes
**Prone position techniques**		
Stimson technique	Patient prone on a stretcher Weights attached to the affected arm hanging off the side of the stretcher Intraarticular anaesthetist required	No
Scapular manipulation	Patient positioned prone Inferior tip of scapula pushed medial and inferior Superomedial portion of the scapula is held stationary	No

*Source:* Please see the full reference list of the article Cunningham NJ. Techniques for reduction of anteroinferior shoulder dislocation. Emerg Med Australas. 2005;17(5–6):463–471. https://doi.org/10.1111/j.1742-6723.2005.00778.x, for more information

Each of these reduction techniques utilises either traction, leverage, manipulation of the humeral head or a combination of these manoeuvres with the affected arm in various positions. Cunningham published a comprehensive review of different techniques available for reducing an anteriorly dislocated shoulder.^7^ The multitude of available reduction techniques speaks to the fact that not one single manoeuvre is successful in all cases.

**FIGURE 1:**
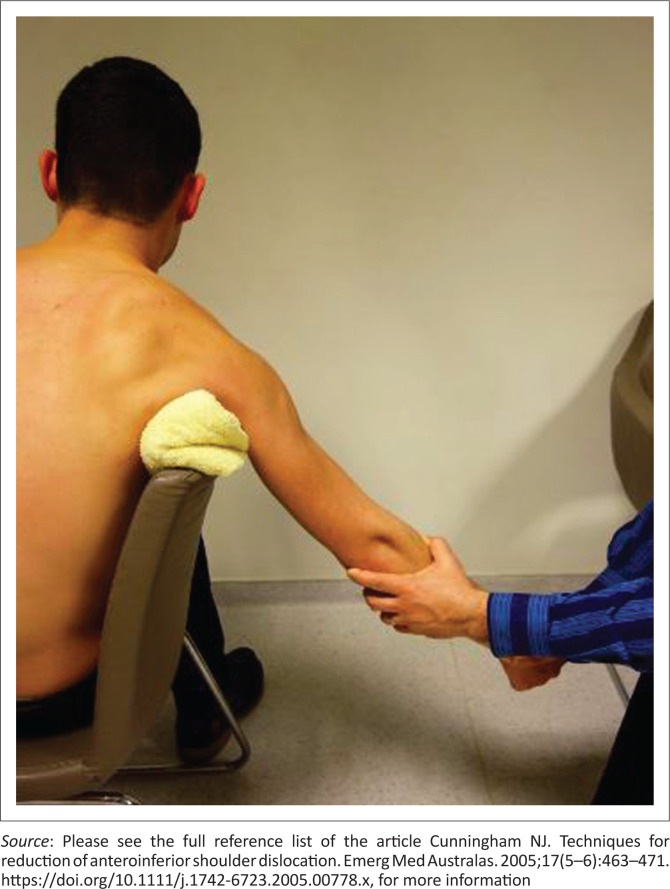
The chair method.

A comparative review of four techniques suggested that the chair method was the easiest to perform and had the fastest reduction time.^9^ In the chair method, the patient is asked to sit on a stable chair sideways. The backrest of the chair is used as a fulcrum in the axilla. The backrest of the chair should be well-padded by a folded bed sheet or small, stiff pillow. This minimises the risks of an axillary nerve injury or iatrogenic fracture. The dislocated arm is allowed to hang over the backrest of the chair. The physician squats down behind the chair, holds the patient’s elbow with the left hand for a right shoulder dislocation and induces the patient’s arm to gently flex at the elbow (Figure 1). The physician’s other hand holds the patient’s right hand still. The patient is asked and encouraged to relax and be calm or distracted with engaging questions; traction is applied slowly by the left hand of the physician, and reduction occurs at this stage. A slight amount of external rotation can be applied by the right hand of the physician to aid in reduction.^11,12^

The reduction is successfully completed when a clunk is heard as the humeral head moves into place.

Care is taken to fully examine the brachial and radial pulses for volume and symmetry to the contralateral side, and to assess the brachial plexus as previously done before the reduction manoeuvre. The incidence of neurological injuries associated with anterior shoulder dislocation is much higher than clinically suspected. De Laat et al. looked at 101 patients with shoulder dislocation and reported that 45% of cases had electromyographic evidence of nerve injuries. The axillary and suprascapular nerves were the most injured nerves.^5^ Other studies have reported rates of neurological injuries varying from 13% to 36%.^13,14^ The reduced shoulder is protected in an arm sling with the arm to the side and the elbow flexed at 90 degrees.

**FIGURE 2:**
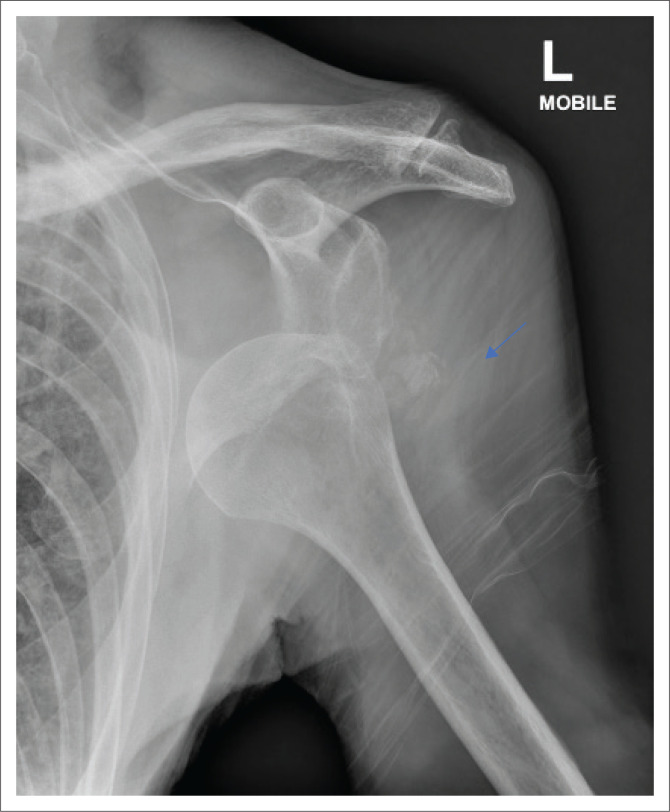
Anterior shoulder dislocation of the left shoulder; faint bony fragments are seen in the region of the greater tuberosity (blue arrow).

**FIGURE 3:**
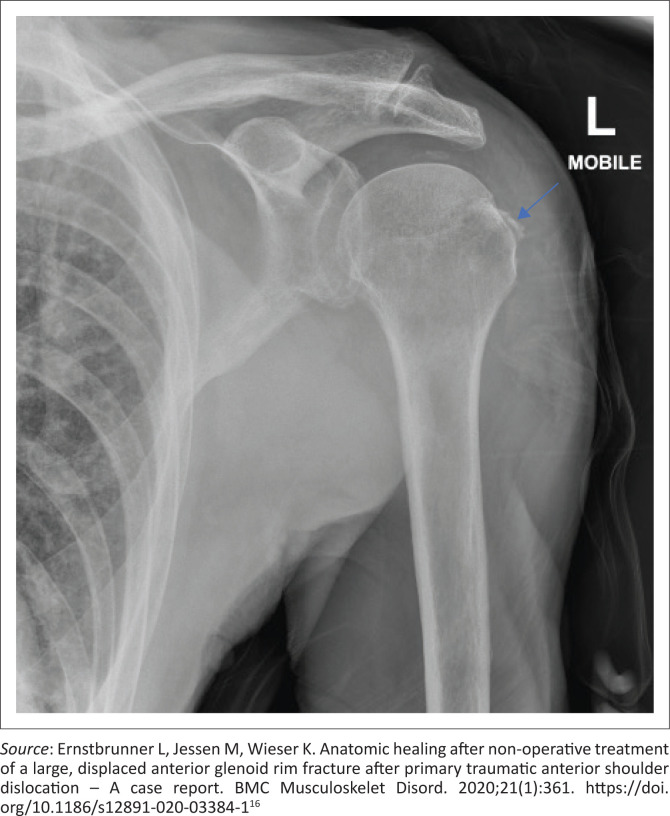
Same shoulder, reduced but in internal rotation, a flake of bony fragment is visible in the greater tuberosity region (blue arrow).

**FIGURE 4:**
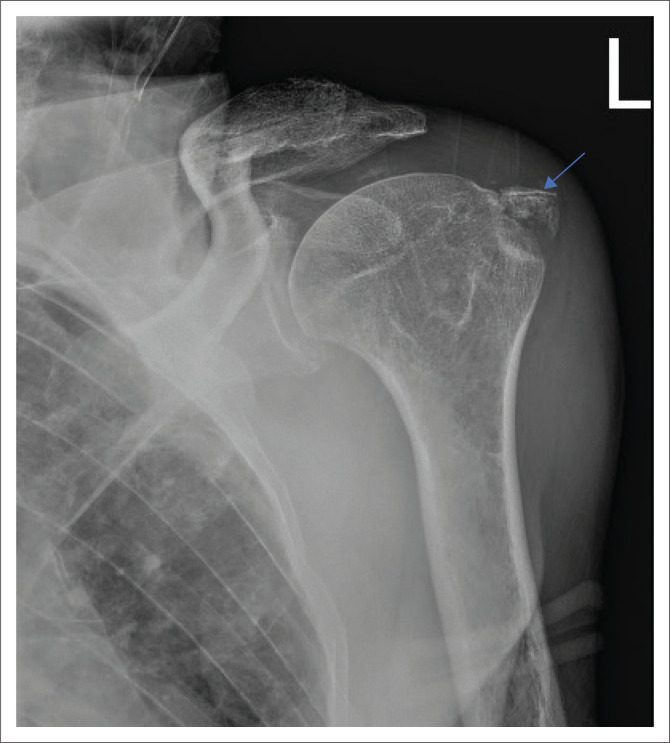
Same shoulder, reduced, but in external rotation, notice the well-defined, displaced greater tuberosity avulsion fracture (blue arrow).

**FIGURE 5:**
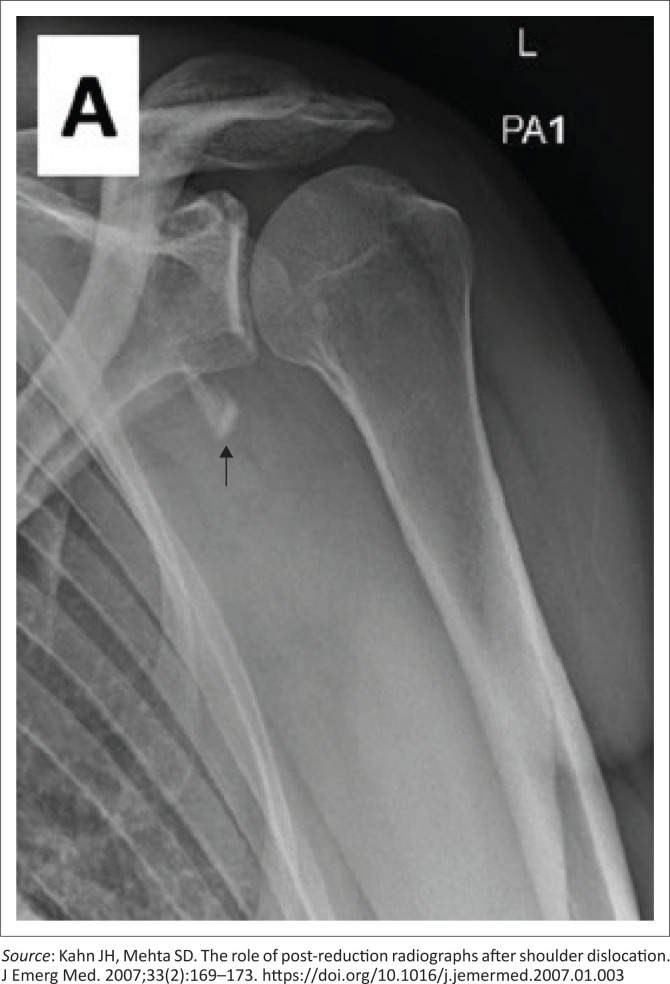
Antero-inferior glenoid rim fracture after anterior glenohumeral dislocation.

Control plain radiographs are mandatory. Three views must be obtained (anteroposterior, axillary view and lateral view) to confirm the reduction and rule out bony compression fractures of the humeral head (Hill-Sachs lesion), push-off fractures of the glenoid (bony Bankart lesion), bony erosions (glenoid bone loss) or to identify fractures that were initially not visible (Figure 2, Figure 3, Figure 4 and Figure 5). Kahn and Mehta reported a rate of 37.5% of fractures which were visible only on post-reduction radiographs.^15^

## Irreducible acute anterior shoulder dislocations

In rare cases, the clinician may not be able to reduce the shoulder after the first or second attempt without sedation. In these cases, a closed reduction manoeuvre should be attempted under appropriate sedation using a method that is feasible with the patient lying supine for better airway control and monitoring of vital signs. If the third attempt under sedation is also unsuccessful, plain radiographs should be repeated to exclude fractures that were initially missed, or iatrogenic fractures, and the patient must be urgently referred to the orthopaedic surgeon for further assessment and surgical relocation of the joint.

**FIGURE 6:**
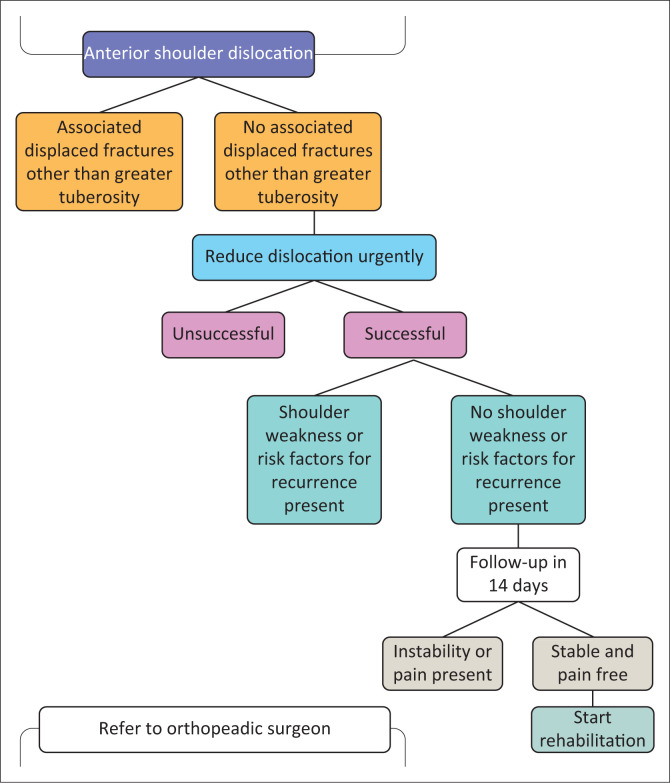
Management algorithm of the first-time acute traumatic anterior shoulder dislocation in primary healthcare.

Successfully reduced first-time anterior dislocations of the shoulder not associated with fractures or shoulder weakness can be successfully managed by the primary health care physician (Figure 6). The subjects of debate at this point in the management are the position in which the arm should be maintained during the immobilisation period, how long should the arm be immobilised for and when can the patient be allowed to return to their full recreational activities or sports activities if they were professional athletes.

## Position of immobilisation

The conventional immobilisation position after reduction of a dislocated shoulder is with the arm to the side of the body and the shoulder internally rotated. In the early 1990s, there was an increased enthusiasm for immobilisation of the shoulder in external rotation with or without abduction. A cadaveric biomechanical study showed that in external rotation the avulsed antero-inferior capsulolabral complex reduces better and is well compressed to the glenoid by the tension in the subscapularis muscle.^17^ These findings were later confirmed in an magnetic resonance imaging (MRI) study that showed that when the shoulder was placed in external rotation, the anterior capsulolabral tissues were less displaced than they were when the shoulder was in internal rotation.^18^ These studies supported the concept of immobilisation in external rotation. However, an arthroscopic study of dislocated shoulders concluded that although external rotation reduced the capsulolabral complex, this reduction was incomplete.^19^ Follow-up studies have not shown a difference in recurrence rates between shoulders immobilised in external rotation versus those immobilised in internal rotation over time.^20^ Immobilisation in external rotation with or without abduction is uncomfortable and less tolerated by patients. As the position of immobilisation after a reduction does not influence the recurrence rate,^21^ the standard immobilisation position should remain that of the arm on the side of the body and the shoulder in internal rotation supported in an arm sling.^22^

## Duration of immobilisation

The duration of immobilisation considers factors such as age of the patient and associated fractures such as greater tuberosity or scapula fractures. Younger patients are prone to recurrent dislocations and for their first dislocation, the immobilisation period may last until they are comfortable to get out of the sling but immobilisation may not be extended beyond 3 weeks. Older patients are prone to rotator cuff tears and shoulder stiffness. Older patients with no rotator cuff tear and no fractures should be immobilised for 10–14 days to allow pain to settle and rehabilitation should begin early to prevent stiffness.^23^ A meta-analysis study found that immobilisation beyond 1 week did not decrease the rate of recurrent shoulder dislocation.^24^ In the presence of a greater tuberosity fracture that is well reduced after the relocation manoeuvre, the shoulder should be immobilised for 4–6 weeks to allow fracture healing. Passive rehabilitation may be started at 4 weeks to prevent stiffness.

## Return to play and full activities of life

This is the most frequently asked question by patients on the day of the injury; however, there is no consensus on a time frame for when patients should be allowed to fully resume activities of life and return to sports activities. The general understanding is that the patient must be pain free and have at least 90% of the range of motion and strength comparative to the uninjured side. This would normally take 2–3 weeks in conservatively treated patients.^25,26^

## Follow-up assessment

Every shoulder dislocation must be reviewed at 10–14 days after the initial incident. At this stage, the pain level is expected to be better, and the patient can be examined for persistent instability, risk factors for a recurrent shoulder dislocation, rotator cuff tears or neurological injuries that could not be picked up at the initial examination.

## Persistent instability

Persistent shoulder pain or guarding on follow-up examination should raise the suspicion for persistent instability. This is confirmed with an apprehension test. In this test, the patient is either seated or lying supine, and the examiner passively abducts and externally rotates the affected shoulder.^27,28^ The test is positive when the patient resists the movement in attempt to prevent a dislocation or reports pain. The test is positive between 45 degrees and 90 degrees of abduction and external rotation. This test’s sensitivity is 50% – 55% and its specificity is 90% – 100%.^29,30^ Patients with a positive apprehension test should be referred to the orthopaedic shoulder specialist for special investigations and assessment of the need for early stabilisation surgery.

## Risk factors for recurrent anterior shoulder dislocation

Patients younger than 20 years of age at the time of the initial dislocation are particularly at an increased risk of a recurrent dislocation.^1^ Other risk factors include generalised ligament laxity as assessed per the Beighton scoring system,^31,32,33^ humeral (Hill–Sachs lesion)^34^ or glenoid bone loss,^35^ and participation in competitive contact sports.^25,36,37^

## Rotator cuff tears

This complication of a shoulder dislocation is seen in patients older than 40 years of age. It is important to make the diagnosis early as a traumatic cuff tear is treated with early surgery for better outcomes.^38^ Patients with a rotator cuff tear will present with weakness or absence of abduction and external rotation for the posterosuperior cuff tear (supraspinatus and infraspinatus tendons) or increased external rotation and weak or absent internal rotation for the subscapularis tendon tears. Once more, point-of-care ultrasound is commonly used by skilled practitioners for the diagnosis of rotator cuff tears.

## Neurological injuries

The axillary and suprascapular nerves are the most commonly injured nerves.^5^ These injuries are often a neuropraxia and true nerve tears are very rare.^22^ A careful assessment of these nerves and a full brachial plexus examination is required at follow-up. At times a clinician may not be able to differentiate between a nerve injury and a rotator cuff tear as both will present with shoulder weakness. Sensory abnormalities and associated absent or weak elbow active flexion will favour a brachial plexus injury. A shoulder point of care ultrasound or special investigations such as MRI and nerve conduction studies will provide a final diagnosis. In certain instances, a patient may present with both a rotator cuff tear and nerve injury in the so-called terrible triad of the shoulder.^22^ These patients should all be referred to an orthopaedic surgeon for specialist care.

## Conclusion

Anterior shoulder dislocation is a common traumatic injury treated in the primary healthcare setting. This narrative has set out an approach to the assessment and management of the first-time acute anterior shoulder dislocation. After the initial clinical and plain radiographs assessment, an ideal method of reduction is employed to relocate the shoulder. Following a successful relocation, a post-reduction assessment is conducted and the decision is made to either follow up and manage the patient in the primary healthcare setting or refer to the specialist depending on the risk of a recurrent dislocation or the presence of associated injuries. Such an approach will ensure that each patient receives individualised treatment in an attempt to address complications early and prevent a recurrent dislocation inherently associated with high risk of shoulder osteoarthritis in a young patient.
